# A Simple and Rapid Microscale Method for Isolating Bacterial Lipopolysaccharides

**DOI:** 10.3390/ijms25126345

**Published:** 2024-06-08

**Authors:** Daniil Grumov, Alexey Kostarnoy, Petya Gancheva, Alexey Kondratev

**Affiliations:** Laboratory of Rickettsial Ecology, N. F. Gamaleya National Research Center of Epidemiology and Microbiology, 123098 Moscow, Russia

**Keywords:** lipopolysaccharides, bacterial endotoxin, inflammation, cell signaling, cytokines, mass spectrometry

## Abstract

Bacterial endotoxins (lipopolysaccharides (LPSs)) are important mediators of inflammatory processes induced by Gram-negative microorganisms. LPSs are the key inducers of septic shock due to a Gram-negative bacterial infection; thus, the structure and functions of LPSs are of specific interest. Often, highly purified bacterial endotoxins must be isolated from small amounts of biological material. Each of the currently available methods for LPS extraction has certain limitations. Herein, we describe a rapid and simple microscale method for extracting LPSs. The method consists of the following steps: ultrasonic destruction of the bacterial material, LPS extraction via heating, LPS purification with organic solvents, and treatment with proteinase K. LPSs that were extracted by using this method contained less than 2–3% protein and 1% total nucleic acid. We also demonstrated the structural integrity of the O-antigen and lipid A via the sodium dodecyl-sulfate polyacrylamide gel electrophoresis (SDS–PAGE) and matrix-assisted laser desorption ionization mass spectrometry (MALDI–MS) methods, respectively. We demonstrated the ability of the extracted LPSs to induce typical secretion of cytokines and chemokines by primary macrophages. Overall, this method may be used to isolate purified LPSs with preserved structures of both the O-antigen and lipid A and unchanged functional activity from small amounts of bacterial biomass.

## 1. Introduction

Bacterial lipopolysaccharides (LPSs) are the major components of the cell envelope of Gram-negative bacteria [1]. In addition to their role as structural components of bacteria, LPSs are bacterial endotoxins that exhibit broad-ranging effects on host cells. Bacterial lipopolysaccharides are amphiphilic molecules that contain hydrophobic components, such as lipid A, and hydrophilic components, such as the core oligosaccharide and polysaccharides, including the O-antigen [2].

Lipid A is a pathogen-associated molecular pattern (PAMP) that includes phosphorylated diglucosamine with variable amounts of groups acylated by fatty acids, and this structure can be recognized by the innate immune receptor Toll-like 4 (TLR4). The number of acylated groups may vary from four to eight. Lipid A can also contain modifications other than phosphorylation [3]. Hexa-acylated and diphosphorylated lipid A isolated from *E. coli* is the most potent agonist of the TLR4 receptor [4]. The recognition of LPSs by TLR4 leads to the induction of the proinflammatory innate immune cascade and the secretion of a broad spectrum of inflammatory mediators, such as cytokines and chemokines. LPS–TLR4 interaction and the subsequent induction of the hyperinflammatory response underlie the pathogenesis of sepsis-associated infection by Gram-negative bacteria [5].

The investigation of the properties and structure of bacterial endotoxins is essential to understanding the pathogenetic processes of TLR4-mediated inflammation; moreover, to perform these studies, pure bacterial lipopolysaccharides must be isolated. Currently, there are many different methods for isolating lipopolysaccharides. Some of these methods are applicable for the large-scale extraction of lipopolysaccharides, including the method described by Westphal and Jann [6], the method described by Galanos et al. [7], and the method described by Darveau and Hancock [8]. However, these methods are difficult to apply when only a small amount of bacterial biomass is available. For example, Lindsay et al. [9] previously showed that the Westphal or Darveau methods may produce a relatively low yield of LPSs. Additionally, to date, several methods that are applicable for the microscale extraction of LPSs have been developed, such as the method of extracting LPSs by boiling [10], the method of extracting LPSs by using phenol and guanidine thiocyanate [11], and the method of extracting LPSs by boiling with SDS and subsequent digestion by proteinase K, which was described by Hitchcock et al. [12]. Unfortunately, the quantitative characterization of impurities (mainly proteins and nucleic acids) in LPSs extracted via these methods has not been thoroughly investigated. Using LPSs with relatively high levels of proteins and nucleic acid impurities in vitro and in vivo can lead to not only LPS–TLR4-mediated inflammation but also TLR2- and TLR9-mediated inflammation.

Herein, we describe a fast and simple microscale method for extracting different types of bacterial endotoxins consisting of the following steps: sonication to disintegrate bacterial aggregates, boiling extraction of LPSs, chloroform–methanol–water extraction, and enzymatic digestion of residual proteins. The structural integrity of the O-antigen after LPS extraction by using this method was demonstrated via electrophoresis in polyacrylamide gel with subsequent silver staining. Additionally, lipid A integrity was demonstrated via mass spectrometry analysis. Moreover, by using primary human macrophages, we showed that the described method produces LPSs with a preserved ability to induce the secretion of cytokines and chemokines specific to LPS–TLR4-mediated inflammation.

## 2. Results

For more than 100 years, the use of bacterial endotoxins (or LPSs) has been an interesting and enigmatic subject of investigation [13]. Despite the widespread use of various methods for LPS extraction, all of the methods are characterized by several limitations, such as the use of reagents that degrade LPSs [14], inapplicability at a low scale [9], and the absence of data regarding the structure of the extracted LPSs and the content of their impurities [10,11]. Additionally, extraction methods are often highly complex and time-consuming.

**Figure 1:**
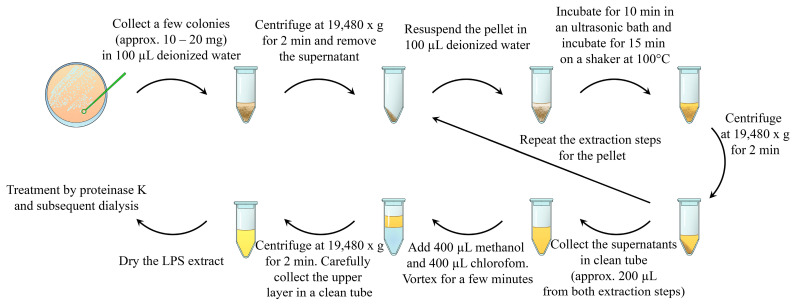
Schematic representation of the described method of bacterial lipopolysaccharide isolation. The bacterial sample (approx. 10–20 mg) was collected in 100 µL of deionized water and centrifuged. The supernatant was discarded. The pellets were resuspended in 100 µL of deionized water and subjected to an ultrasonic bath. After sonication, LPSs were extracted by heating the suspension. Water LPS extract and cell debris were separated by using centrifugation. The supernatant was collected in a clean tube, and LPS extraction from the pellets was repeated via resuspension, sonication, and heating. The prepared water LPS extracts were purified by using chloroform–methanol–water extraction and enzymatic treatment with proteinase K.

Herein, we describe a rapid and simple method for the extraction of purified LPSs. The method consists of the following steps: ultrasound destruction of bacterial aggregates, as well as bacterial cells; LPS release from the bacterial cell wall using heating [15,16]; LPS extraction using volatile and easily removable organic solvents; and enzymatic treatment with proteinase K (Figure 1).

Our experiments were performed on cultures of *Pseudomonas aeruginosa* PA103 and *Escherichia coli* ATCC 25922 strains, which are widely used as typical Gram-negative bacteria. The bacterial strain *E. coli* ATCC 25922 was selected for additional culture to demonstrate the ability of the described method to extract LPSs from different Gram-negative bacteria. We used bacteria in the stationary growth phase cultivated in solid culture media for our experiments. To evaluate the structural integrity and biological activity of LPSs obtained via the described method, we used LPSs extracted via the Westphal method, which is a widely known “gold standard” of LPS extraction, as a control [6]. The principles of the Westphal method are similar to those of the method described in this study, as both are based on the heating of the bacterial culture, followed by the release of LPSs and extraction of the substances based on hydrophobic properties. The extracts that were prepared by using both methods were fully dried in a vacuum evaporator and stored at −20 °C until the subsequent analysis of yield, percentage of impurities, structural integrity, and biological activity. LPSs isolated via the Westphal method were subjected to dialysis against water to remove residual phenols.

### 2.1. Distribution of LPSs during Extraction Procedure

In the first stage of our experiments, we investigated the distribution of LPSs in the layers of the chloroform–methanol–water extraction mixture and selected the layer containing the maximal amount of LPSs. For this purpose, we collected each layer separately, dried it in a vacuum concentrator, resuspended it in deionized water, and analyzed it by using the widely applicable Limulus Amebocyte Lysate test (LAL test) [17]. The LAL test results demonstrating the distribution of LPSs in the layers are presented in Table 1. As the methanol–water layer contained the greatest amount of LPSs, further experiments were performed with only this layer. 

### 2.2. Characterization of Yield and Purity

Subsequently, we analyzed the yield of the product and impurities. To evaluate the yield, we quantified the amount of LPSs by using the LAL test. The yield was calculated as the normalized endotoxin activity per unit wet weight of bacterial culture used for extraction (Table 2).

We also evaluated the content of coextracted protein and nucleic acid impurities, as both proteins and nucleic acids were previously described as ligands of innate immune receptors [18]. To quantify the nucleic acid content, we used highly sensitive kits for RNA and DNA quantification via a Qubit 3.0 system. Proteins were also quantified by using the Qubit 3.0 system. LPSs isolated from *E. coli* and *P. aeruginosa* cultures by using the described method without enzymatic treatment contained approximately 20% protein (Table 3), whereas the total level of DNA and RNA was approximately 1–1.5%. Therefore, we performed enzymatic treatment of the extraction product with proteinase K to remove protein impurities. After incubation with proteinase K, residual proteins and proteinase K were denatured via heating and removed by using centrifugation. After enzymatic digestion, residual peptides were removed via dialysis through a membrane with a cutoff of 3 kDa against deionized water. Both the protein and nucleic acid contents in the obtained extracts were again measured. According to the data presented in Table 3, after enzymatic treatment, the LPSs that were isolated by using the described method contained low levels of nucleic acids and proteins. Additionally, we recommend the use of enzymatic digestion with nucleases to obtain purer LPSs.

### 2.3. Analysis of O-Antigen’s Composition

**Figure 2:**
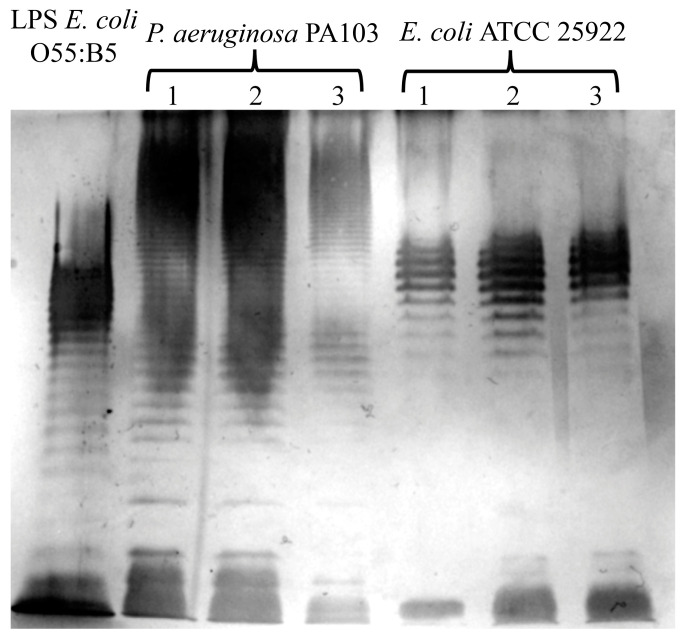
The evaluation of O-antigen integrity via electrophoresis in polyacrylamide gel and the subsequent silver staining of LPSs extracted via the herein described method from *P. aeruginosa* PA103 and *E. coli* ATCC 25922. Typical O-antigen patterns for these bacteria are shown. The extraction was repeated three times, and the products from each extraction were individually subjected to SDS–PAGE. Commercially available LPS from *E. coli* O55:B5 was used as a control.

We evaluated the effects of extraction by using the described method on the structure of LPSs. To analyze the structural integrity of the O-antigen, we visualized LPSs via electrophoresis in 12% polyacrylamide gel and subsequent silver staining [19]. A photograph of the silver-stained gel obtained after the extract that was prepared using the described method underwent electrophoresis is presented in Figure 2. Commercially available LPSs from the *E. coli* 055:B5 strain were used as a control. We observed typical LPS profiles of *E. coli* ATCC 25922 [20] and *P. aeruginosa* PA103, as described previously [21]. These data indicate that the described method of extraction did not affect the O-antigen structure.

### 2.4. Structural Analysis of Lipid A

**Figure 3:**
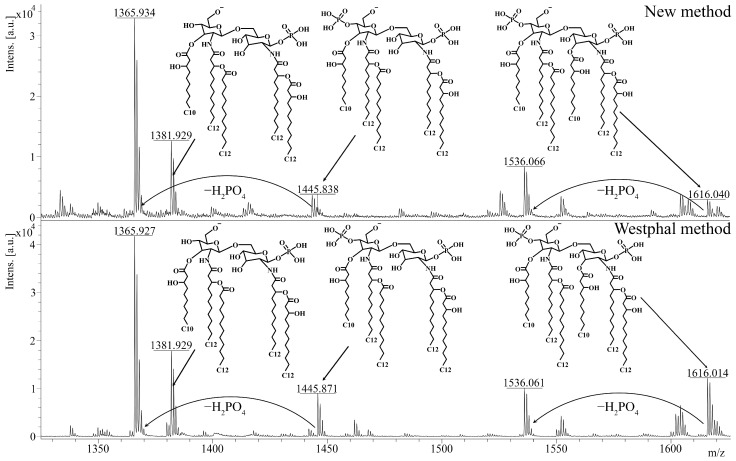
The analysis of lipid A via mass spectrometry. The spectra of lipid A from LPSs extracted from *P. aeruginosa* PA103 by using the described method and the Westphal method are presented. Peaks with *m*/*z* = 1616 correspond to hexa-acylated diphosphorylated lipid A, whereas peaks with *m*/*z* = 1536 correspond to hexa-acylated monophosphorylated lipid A. Diphosphorylated penta-acylated lipid A has a peak at *m*/*z* = 1446, and monophosphorylated penta-acylated lipid A contains ions with a peak at *m*/*z* = 1382 or *m*/*z* = 1366. The analysis was performed on negative ions by using MALDI-TOF-TOF UltrafleXtreme. *m*/*z*, mass-to-charge ratio.

Afterwards, we investigated the effect of the described method on the chemical structure of lipid A. As lipid A determines the ability of LPSs to interact with the TLR4 receptor, this analysis was of specific importance for evaluating the quality of the extraction. To obtain lipid A, we applied a previously described method of acidic hydrolysis [22] to the extracts that were isolated from *P. aeruginosa* PA103 by using the described method and the Westphal method. The polysaccharide residue and lipid A were separated via ultracentrifugation. Lipid A was dried in a vacuum evaporator and diluted in a chloroform–methanol–water mixture (4:4:1, *v*/*v*/*v*) for further mass spectrometry. The structure of lipid A was analyzed by using the MALDI-TOF-TOF UltrafleXtreme system and 2,6-dihydroxyacetophenone as a matrix (10 mg/mL). Afterward, we compared the mass spectra of the isolated lipid A to study the effects of extraction on the structure of lipid A. Unsurprisingly, the spectra of lipid A from the LPSs that were isolated by using the described method and the Westphal method were very similar (Figure 3). In both spectra, the ions with *m*/*z* = 1616 and *m*/*z* = 1536 corresponded to hexa-acylated di- and monophosphorylated lipid A, respectively, and the ions with *m*/*z* = 1446, *m*/*z* = 1382, and *m*/*z* = 1366 corresponded to penta-acylated lipid A with various degrees of phosphorylation. In accordance with the literature [23], these structures are typical for the *P. aeruginosa* PA103 strain. Therefore, the described extraction method did not likely affect the structure or the ability of lipid A to induce inflammation.

### 2.5. Analysis of Biological Activity of LPSs

**Figure 4:**
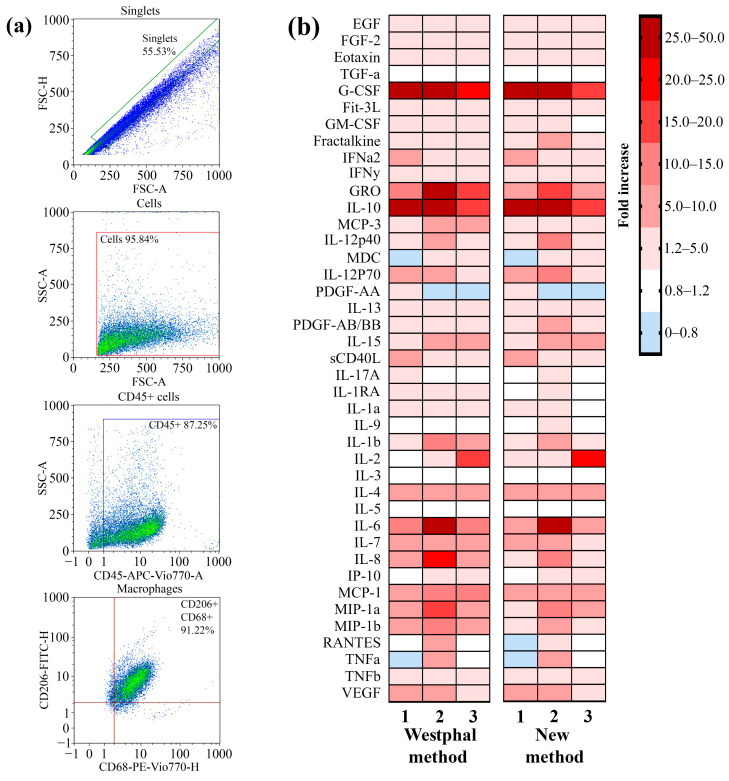
A comparison of the ability of LPSs extracted by using the described method and the Westphal method to induce the secretion of proinflammatory cytokines and chemokines by cultured primary macrophages. (**a**) Gating strategy for analyses of primary macrophages before LPS treatment. An FSC-H vs. FSC-A plot was used to exclude doublets or larger aggregates, and cells in this gate were further analyzed with an SSC-A vs. FSC-A dot plot to identify the original total cell population. The gated population was further analyzed for the expression of CD45, and subsequent analysis were performed with the population of CD45+ cells for CD68 and CD206 expression. (**b**) A heatmap showing the fold increase in the secretion of cytokines and chemokines after the exposure of primary macrophages to the extracted LPSs compared with a nontreated culture.

The ability of the LPSs that were extracted by using the described method to induce the secretion of cytokines and chemokines as a result of TLR4–LPS interaction by primary macrophages was analyzed. Primary macrophages were differentiated from peripheral blood mononuclear cells (PBMCs) that were isolated from the blood of three volunteers by using the GM-CSF differentiation factor. To verify the phenotype, the macrophages were stained with fluorescent antibodies against specific markers for macrophages, such as CD45, CD68, and CD206. By using flow cytometry, we demonstrated that 91% of the cell population possessed the CD45 + CD68 + CD206 + phenotype (Figure 4a). Primary macrophages were incubated with LPSs from *P. aeruginosa* strain PA103 extracted by using the described method and the Westphal method for 24 h. Macrophages not treated with LPSs were used as a control. After incubation, the concentrations of the secreted cytokines and chemokines in the cell media were measured by using a kit for the multiplex analysis of cytokines and chemokines. A heatmap showing an increase in the secretion of cytokines and chemokines in LPS-treated primary macrophages compared with nontreated cells is shown in Figure 4b. We observed that the LPSs extracted via both methods induced similar patterns of secretion of mediators that were specific to TLR4–LPS interaction-associated inflammation. Hence, the method described in this study for LPS extraction did not affect the ability of LPSs to induce inflammation.

## 3. Discussion

The main advantage of the described method in this study is the ability to extract intact LPSs with high purity from a small amount of bacterial biomass without long-term accumulative cultivation. Thus, this micromethod is intended to be useful for extraction from pure isolated cultures that are difficult to cultivate, such as strains with low growth rates, bacteria-producing biofilms, or anaerobic bacteria (for example, members of the intestinal microbiome, such as *Akkermansia*, *Bacteroides*, *Prevotella* [24], and some others).

Herein, we described a method of extraction from a solid culture medium in which purified LPSs were isolated. However, the possibility of LPS extraction from obligate intracellular parasites, such as *Rickettsia* spp. (which are cultivated in eukaryotic cell cultures), should be further investigated. The described method is characterized by simplicity and a high rate of extraction. LPS extraction via the Westphal method normally takes approximately 7 days, whereas pure LPSs can be isolated from bacterial cultures by using the described method within 2 days. The described method uses chloroform and methanol for extraction, which can be easily removed under vacuum for 1–2 h. Alternately, conventional methods usually require 2–3 days of dialysis to remove phenol or SDS. 

Furthermore, small-scale extraction is of specific importance due to the significant variability in LPS synthesis processes in some bacteria during infection or cultivation. In particular, variability in the structure of the O-antigen or lipid A occurs in various microorganisms during chronic inflammatory processes. Chronic infection during cystic fibrosis is associated with a change in the structure of the O-antigen in *P. aeruginosa* from smooth to rough [25], whereas changes in the lipid A structure diminish the ability of LPSs to interact with TLR4 [26]. Correspondingly, the structure of the O-antigen of *Helicobacter pylori* during chronic gastric ulcers may change due to the addition of fucose to the polysaccharide residue, and the compound then mimics human Lewis antigens Le^a^ and Le^b^ [27,28]. Similar variability was observed in *Coxiella burnetii*, in which the number of repeats of the O-antigen polymer in its structure changed during the transition of the inflammatory process from the acute to the chronic stages [29]. The conditions of cultivation on culture media may also affect LPS structure. For example, changes in cultivation temperature can lead to changes in the structure of the O-antigen of *Bordetella parapertussis* [30]. Additionally, the lipid A structure may be affected by changes in the composition of the culture medium [31]. Consequently, small-scale extraction of LPSs by using micromethods provides an opportunity to capture the “fingerprint” variability of LPSs by minimizing the effects of the cultivation steps. We showed that the described microscale method may be used for these purposes because (i) it allows for the isolation of LPSs with preserved structures of the O-antigen and lipid A and (ii) the extracted LPSs can induce a specific TLR4–LPS-induced inflammatory response. Thus, the described method could be widely used to control the variability in LPS structure and properties.

## 4. Materials and Methods

### 4.1. Bacterial Strains and Growth Conditions

The *P. aeruginosa* strain PA103 and the *E. coli* strain ATCC 25922 were obtained from the collection of the N. F. Gamaleya National Research Center of Epidemiology and Microbiology. The *P. aeruginosa* strain PA103 and the *E. coli* strain ATCC 25922 were grown on plates with LB agar medium (FBIS SRCAMB, Obolensk, Russia) at 37 °C for 48 h.

### 4.2. Procedure for Extracting LPSs by Described Method

The scheme for the method is shown in Figure 1. Approximately 10–20 mg wet weight of bacterial cells of each strain was harvested from agar plates by using a bacterial loop and mixed with 100 µL of deionized water from Milli-Q Advantage A10 system (Merck, Darmstadt, Germany). The resulting suspensions were washed via centrifugation at 19,480× *g* for 2 min, after which the supernatant was discarded. The pellet was resuspended in 100 µL of deionized water. The bacterial suspensions were ultrasonicated in an Elmasonic S30H (Elma Schmidbauer GmbH, Singen, Germany) ultrasonic bath for 10 min at room temperature and a working frequency of 37 kHz. Afterwards, the suspensions were incubated in a shaker at 100 °C for 15 min and centrifuged at 19,480× *g* for 2 min. The supernatant or water extract was collected in another tube; the pellet was resuspended in 100 µL of deionized water and treated as described above, starting from the stage of the ultrasonication bath. Both acquired water extracts were combined in one tube. Two hundred microliters of each water extract was subjected to chloroform–methanol–water extraction (both organic solvents from Merck, Darmstadt, Germany) in a ratio of 2:2:1 (*v*/*v*/*v*).The extraction mixture was vortexed for 1 min and centrifuged at 19,480× *g* for 2 min. The upper layer represented the LPS-rich fraction. The upper layer was collected in a clean tube and dried by using a vacuum concentrator CentriVap (Labconco Corporation, Kansas city, MO, USA). The dried LPS extracts were resuspended in a 1 mL solution of 1 µg/mL proteinase K (Sigma-Aldrich, Saint Louis, MO, USA) in a buffer that contained 10 mM Tris-HCl (pH: 7.5; Thermo Scientific, Rockford, IL, USA) and 2 mM CaCl_2_ (Merck, Darmstadt, Germany). The samples were incubated overnight at 37 °C. The enzymes were inactivated via incubation for 15 min at 90 °C. Afterwards, the samples were centrifuged at 12,000× *g* for 10 min. The supernatant was collected in a clean tube and subjected to dialysis through a dialysis membrane with a cutoff of 3 kDa (Thermo Scientific, Rockford, IL, USA) against deionized water overnight. After dialysis, the LPS-containing extracts were dried by using a vacuum concentrator CentriVap (Labconco Corporation, Kansas city, MO, USA). The dried samples were stored at −20 °C until further analysis.

### 4.3. Westphal Method

We used the widely known method of extracting lipopolysaccharides described by Westphal and Jann as a control method [6]. Briefly, we subjected approximately 250–500 mg of wet weight of bacterial cells of each strain to inactivation via acetone (Applichem, Darmstadt, Germany) and drying. Dried and inactivated bacterial cells were treated at 69 °C in a phenol–water mixture (45:55, *v*/*v*) for 15 min. After cooling and centrifugation at 5000× *g* for 30 min, the upper water layer was collected in a clean tube. The lower phenol layer was again subjected to extraction by adding an equal volume of water and treated as described above. Both upper water layers were combined and subjected to dialysis through a dialysis membrane with a cutoff of 3 kDa (Thermo Scientific, Rockford, IL, USA) against deionized water for 1 day to remove trace phenol. After dialysis, the water extract was dried in a vacuum concentrator CentriVap (Labconco Corporation, Kansas city, MO, USA). The dried samples were stored at −20 °C until further analysis.

### 4.4. Isolation of Lipid A

Lipid A was obtained by using the mild hydrolysis method described by Shaw and Hodder [22], with some modifications. Lipid A was isolated from lipopolysaccharides by using a previously described method and by using the Westphal method. Briefly, the dried LPS extracts were dissolved in 1 mL of a 2% (*v*/*v*) acetic acid solution (Applichem, Darmstadt, Germany) and incubated for 2 h at 100 °C. Insoluble lipid A was separated by using an Optima L-90K Ultracentrifuge (Beckman Coulter, Fullerton, CA, USA) equipped with an SW41Ti rotor at 280,000× *g* for 45 min at 4 °C. The lipid A pellet was resuspended in 1 mL of a mixture of chloroform–methanol–water (4:4:1, *v*/*v/v*). The suspension was transferred to a clean tube and dried on a vacuum concentrator CentriVap (Labconco Corporation, Kansas city, MO, USA). Dried lipid A was stored at −20 °C until further analysis.

### 4.5. Impurity Measurement

The nucleic acids (DNA and RNA) and protein impurities in the extracts were analyzed according to the manufacturer’s instructions by using a QuDye dsDNA High-Sensitivity Assay Kit (Lumiprobe, Moscow, Russia), a Qubit RNA High-Sensitivity Assay Kit (Invitrogen, Eugene, OR, USA), and a QuDye Protein Quantification Kit (Lumiprobe, Moscow, Russia), respectively. The analysis was performed by using a Qubit 3.0 fluorimeter (Invitrogen, Eugene, OR, USA) according to the manufacturer’s instructions.

### 4.6. Limulus Amebocyte Lysate (LAL) Test

The quantification of LPSs in the extracts was determined by using the LAL assay (Lonza Inc., Basel, Switzerland) according to the manufacturer’s instructions. The experiments and data analysis were performed by using an ELx808 microplate reader (BioTek, Winooski, VT, USA) equipped with WinKQCL version 5.3.3. software (Lonza Inc., Walkersville, MD, USA).

### 4.7. SDS–PAGE and LPS Silver Staining

The LPSs in the extracts were visualized according to the method described by Zhu et al. [19], with minor modifications. Briefly, all of the LPS-containing extract solutions and a solution of LPSs from *E. coli* 055:B5 (Sigma-Aldrich, Saint Louis, MO, USA), which was used as a marker for SDS–PAGE, were treated at 80 °C for 30 min and vortexed. The concentration of LPSs in all of the solutions subjected to SDS–PAGE was 1 µg/µL (or 7.5 µg per gel track). Subsequently, the LPS solutions were mixed 1:1 (*v*/*v*) with 2× Laemmli sample buffer (Sigma-Aldrich, Saint Louis, MO, USA), incubated for 5 min at 100 °C and fractionated on SDS-polyacrylamide gels (10 cm by 10 cm by 0.75 mm) containing 4% and 12% acrylamide (Sigma-Aldrich, Saint Louis, MO, USA) in the stacking and separating gels, respectively. Electrophoresis was performed at a constant current of 30 mA. After electrophoresis, the gels were oxidized in 100 mL of fixing solution with 0.7% (*w*/*v*) periodic acid (Sigma-Aldrich, Saint Louis, MO, USA) in 30% (*v*/*v*) ethanol (Merck, Darmstadt, Germany) and 10% (*v*/*v*) acetic acid (Applichem, Darmstadt, Germany) for 10 min. After oxidation/fixation, the gels were washed twice in 100 mL of deionized water for 5 min, subjected to 100 mL of 0.2% (*w*/*v*) silver nitrate (Applichem, Darmstadt, Germany) solution, washed twice with deionized water for 20 s, and impregnated in 100 mL of a solution of 3%(*w*/*v*) sodium carbonate (Applichem, Darmstadt, Germany), 0.02% (*w*/*v*) ascorbic acid (Sigma-Aldrich, Saint Louis, MO, USA), 0.04% (*w*/*v*) sodium thiosulfate (Sigma-Aldrich, Saint Louis, MO, USA), and 0.05% (*w*/*v*) sodium hydroxide (Sigma-Aldrich, Saint Louis, MO, USA) for several minutes to develop images. After the reduction reaction, the gels were subjected to 10% (*v*/*v*) acetic acid solution to stop image development. Gels with silver-stained LPSs were evaluated by using the Gel Doc EZ system (Bio-Rad, Hercules, CA, USA).

### 4.8. Matrix-Assisted Laser Desorption/Ionization Negative Ion Mass Spectrometry

Analyses were performed by using a MALDI-TOF-TOF mass spectrometer UltrafleXtreme (Bruker Daltonics, Bremen, Germany) in negative-ion mode with a mass range of 700–4500 *m*/*z*. The data were acquired in reflectron mode with ion source 1 set to 18 kV, ion source 2 set to 16.335 kV, and a lens voltage of 5.4 kV. The reflector voltages were set to 19.15 kV and 9.688 kV for Reflectors 1 and 2, respectively. Calibration of the mass spectrometer was performed by using Peptide Calibration Standard II (Bruker Daltonics, Bremen, Germany). Lipid A, which was obtained via mild hydrolysis of LPSs extracted by both methods, was dissolved in a mixture of chloroform–methanol–water (4:4:1, *v*/*v/v*). The matrix solution was prepared by dissolving 10 mg of 2,6-dihydroxyacetophenone (Sigma-Aldrich, Saint Louis, MO, USA) in 1 mL of a mixture of acetonitrile (Merck, Darmstadt, Germany) and water (1:1, *v*/*v*). The samples were spotted by mixing 1 µL of the sample drop by drop with 1 µL of matrix solution on a stainless-steel target.

### 4.9. PBMC Isolation and Differentiation

Blood was obtained from three blood donors. All of the blood donors provided written informed consent, and the study was approved by the Bioethics Committee of N. F. Gamaleya National Research Center of Epidemiology and Microbiology. Blood sample collection was performed according to the Declaration of Helsinki principles. The isolation and differentiation of peripheral blood mononuclear cells (PBMCs) were performed according to a widely used protocol, as described previously [32], with some modifications. Briefly, PBMCs were isolated from the donated blood of three healthy blood donors via Ficoll–Hypaque density gradient centrifugation. The cells were then cultured in RPMI medium (Gibco, Paisley, UK) supplemented with 20 nM GM-CSF (Sigma-Aldrich, Saint Louis, MO, USA), 10% (*v*/*v*) foetal bovine serum (HyClone, Logan, UT, USA), 2 mM stable glutamine, 1 mM sodium pyruvate, MEM nonessential amino acids, and an antibiotic/antimycotic solution (all from Capricorn Scientific, Ebsdorfergrund, Germany). The cells were seeded into either 24-well plates or 96-well plates (Corning, Corning, NY, USA). Nonadherent cells were removed after 2 h of incubation at 37 °C in a humidified atmosphere containing 5% CO_2_. The cells were differentiated at 37 °C in a humidified atmosphere containing 5% CO_2_ for 10 days. The medium was changed every 2 days.

### 4.10. Flow Cytometry

Flow cytometry analysis of the primary macrophages was performed by using an MACQuant Analyzer 10 flow cytometer (Miltenyi Biotec, Bergisch Gladbach, Germany) equipped with three laser excitation sources (405, 488, and 635 nm), and the data were evaluated by using MACQuantify V2.11.1817.19623 software (Miltenyi Biotec, Bergisch Gladbach, Germany). Staining was performed with the following fluorochrome-conjugated antibodies: anti-human CD206-VioBlue, anti-human CD86-FITC, anti-human CD45-APC-Vio770, and anti-human CD14-VioGreen (all from Miltenyi Biotec, Bergisch Gladbach, Germany). The cells were labeled with antibodies for 10 min at +4 °C and washed in phosphate-buffered saline. The filter configurations for the measurements were 450/50 nm (VioBlue), 525/50 nm (VioGreen), and 525/50 nm (FITC). A forward scatter threshold was applied to eliminate electronic noise and particles from the flow cytometric data.

### 4.11. LPS Stimulation of Macrophages

The macrophages were cultured in RPMI medium (Gibco, Paisley, UK) supplemented with 20 nM GM-CSF (Sigma–Aldrich, Saint Louis, MO, USA), 10% (*v*/*v*) foetal bovine serum (HyClone, Logan, UT, USA), 2 mM stable glutamine, 1 mM sodium pyruvate, MEM nonessential amino acids, and an antibiotic/antimycotic solution (all from Capricorn Scientific, Ebsdorfergrund, Germany) supplemented with LPSs for 24 h. We used the LPSs from *Pseudomonas aeruginosa* PA103 that were obtained via the described method or via the Westphal method, and the final concentrations of LPSs in the media were 1 and 10 µg/mL. Each treatment for each donor was performed in duplicate.

### 4.12. Quantification of Cytokines and Chemokines

The levels of 41 cytokines and chemokines in the supernatants of LPS-treated and untreated primary human macrophages were measured in duplicate by using the MILLI-PLEX MAP Human Cytokine/Chemokine Magnetic Bead Panel Kit (Merck, Darmstadt, Germany) according to the manufacturer’s instructions. The quantification of cytokines and chemokines was performed by using the MAGPIX Instrument (Luminex Corp., Austin, TX, USA). Data visualization was performed by using GraphPad Prism version 8.0.0 for Windows (GraphPad Software, San Diego, CA, USA).

### 4.13. Statistical Processing

The statistical processing of the data was performed by using Excel 2016 software (Microsoft, Albuquerque, NM, USA).

## Figures and Tables

**Table 1:** The layer distribution of LPSs during extraction with the chloroform–methanol–water mixture. The results obtained by using the LAL test from two independent experiments are presented. The data are shown as the means ± SD.

Strain	Layer	Total Endotoxin Activity per Layer, EU
*P. aeruginosa* PA103	Water–methanol	4.07 × 10^5^ ± 1.43 × 10^5^
	Chloroform–methanol	9.96 × 10^2^ ± 9.66 × 10^2^
*E. coli* ATCC 25922	Water–methanol	6.06 × 10^5^ ± 3.67 × 10^5^
	Chloroform–methanol	2.48 × 10^4^ ± 3.05 × 10^4^

**Table 2:** The normalized endotoxin activity (EU) of the LPS extracts in 1 mg of wet weight bacterial culture. The results that were obtained by using the LAL test for two independent experiments are presented. The data are shown as the means ± SD.

Strain	Wet Mass, mg	EU per 1 mg of Wet Weight of Bacterial Culture
*P. aeruginosa* PA103	13.18 ± 2.58	3.04 × 10^4^ ± 4.92 × 10^3^
*E. coli* ATCC 25922	20.74 ± 0.48	3.48 × 10^4^ ± 1.91 × 10^4^

**Table 3:** Protein and nucleic acid contents in LPSs extracted by using the described method before and after enzymatic treatment with proteinase K. Data from three independent experiments are presented. The data are shown as the means ± SD.

Strain	Protein in Extract (Weight % of Total)		DNA in Extract (Weight % of Total)		RNA in Extract (Weight % of Total)	
	Before	After	Before	After	Before	After
* P. aeruginosa * PA103	19.08 ± 4.65	3.62 ± 1.63	0.92 ± 0.04	0.67 ± 0.07	0.73 ± 0.02	0.78 ± 0.04
* E. coli * ATCC 25922	19.31 ± 0.69	2.33 ± 0.26	0.53 ± 0.02	0.43 ± 0.17	0.63 ± 0.17	0.47 ± 0.02

## Data Availability

All data generated or analyzed during this study are included in this published article.
